# Transparent reporting of hypotheses and analyses in behavioral medicine research: an audit of publications in 2018 and 2008

**DOI:** 10.1080/21642850.2021.1907186

**Published:** 2021-04-07

**Authors:** Megan A. McVay, Kellie B. Cooper, Montserrat Carrera Seoane, Marissa L. Donahue, Laura D. Scherer

**Affiliations:** a Department of Health Education and Behavior, College of Health and Human Performance, University of Florida, Gainesville, FL, USA; b Department of Psychology, Utah State University, Logan, UT, USA; c School of Medicine, University of Colorado, Denver, CO, USA

**Keywords:** Open science, research methods, scientific publication, pre-registration

## Abstract

**Objective:**

We aimed to document the use of transparent reporting of hypotheses and analyses in behavioral medicine journals in 2018 and 2008. **Design:** We examined a randomly selected portion of articles published in 2018 and 2008 by behavioral medicine journals with the highest impact factor, excluding manuscripts that were reviews or purely descriptive. **Main Outcome Measures:** We coded whether articles explicitly stated if the hypotheses/outcomes/analyses were primary or secondary; if study was registered/pre-registered; if ‘exploratory’ or a related term was used to describe analyses/aims; and if power analyses were reported. **Results:** We coded 162 manuscripts published in 2018 (87% observational and 12% experimental). Sixteen percent were explicit in describing hypotheses/outcomes/analyses as primary or secondary, 51% appeared to report secondary hypotheses/outcomes/analyses but did not use term ‘secondary,’ and 33% were unclear. Registration occurred in 14% of studies, but 91% did not report which analyses were registered. ‘Exploratory’ or related term was used in 31% of studies. Power analyses were reported in 8% of studies. Compared to 2008 (*n* = 120), studies published in 2018 were more likely to be registered and less likely to be unclear if outcomes were primary or secondary. **Conclusions:** Behavioral medicine stakeholders should consider strategies to increase clarity of reporting, and particularly details that will inform readers if analyses were pre-planned or post-hoc.

**Study registration:**

https://osf.io/39ztn

## Introduction

In response to concerns about reproducibility and rigor, scientific communities are increasingly focused on improving the transparency of the scientific enterprise (National Academies of Sciences, 2018). Researchers in behavioral medicine and associated fields have been among those calling for greater transparency in research practices (Hagger, 2019; Jago & van der Ploeg, 2018; McVay & Conroy, 2019; Munafò, 2017). This call for transparency is part of a broader movement for a range of scientific research and reporting reforms that seek to improve the reliability of the scientific literature. These reforms include (but are not limited to): (1) open sharing of study materials, data, and analysis code, (2) conducting a priori power analyses and conducting studies with adequate statistical power, (3) pre-registering intended samples sizes, primary outcome measures, statistical analyses, and (4) transparently reporting analyses, such as distinguishing pre-planned and post-hoc analyses (Shrout & Rodgers, 2018).

Reporting transparency is a key aspect of improving the reliability of the scientific literature. Transparency is particularly important in reporting whether the analyses presented were pre-planned or post-hoc. Understanding the context of presented analyses can help readers evaluate the risk of Type I or Type II error inflation in null hypothesis significance testing. Unlike a priori analyses, post-hoc analyses may have been influenced by what was observed during data collection or during data analyses, and thus risk inflating the false positive error rate (Wagenmakers, Wetzels, Borsboom, van der Maas, & Kievit, 2012). With alpha set at 5%, one would expect to observe one false positive result out of every 20 tests. Hence, post-hoc analyses, when presented without statistical adjustment or qualifications, can inflate the false positive rate, and in combination with publication bias that favors significant results, may undermine some scientific literatures (Wagenmakers et al., 2012). Importantly, this can occur in the absence of any malicious intent on the part of authors or publishers, who are simply seeking to report and publish the most interesting and promising findings.

In behavioral medicine research, it is common for a single data collection effort to result in multiple separate publications, a practice which allows data collection efforts to be maximally informative. However, indication of pre-planned analyses and post-hoc analyses is not always evident in published manuscripts. In some cases, specific terms in manuscripts can suggest that analyses presented are post-hoc. In particular, the descriptor *secondary* or *exploratory* may suggest a post-hoc analysis. However, neither of these terms can be assumed to indicate that an analysis was post-hoc; exploratory analyses may be pre-planned but described as exploratory due to a lack of directional hypothesis, and secondary analyses may be pre-planned but secondary to the primary planned analyses. Notably, scientific reporting guidelines recommend making distinctions that can help inform the extent to which analyses might be pre-planned or post-hoc. For example, clinical trials reporting guidelines recommend that authors include in their manuscript a description of ‘completely defined pre-specified primary and secondary outcome measures’ (Schulz, Altman, & Moher, 2010). Reporting guidelines for observational research recommend that authors ‘state specific objectives, including any pre-specified hypotheses’ (von Elm et al., 2008). Similarly, journal article reporting recommendations endorsed by the American Psychological Association (APA) recommend inclusion of ‘primary and secondary hypotheses’ for all quantitative studies (Appelbaum et al., 2018). Individual journals have also focused on this; for example, PLOS Medicine requests that observational studies clearly report which hypotheses authors intended to test, and how and why what was reported differed from what was planned (PLOS Medicine Editors, 2014).

Clarity of which analyses presented were pre-planned and which were post-hoc can be facilitated by pre-registering an analysis plan – documentation of what analyses researchers intend to conduct, placed in a publicly available, time-stamped repository prior to data collection (or in some cases, prior to examining data; Nosek, Ebersole, DeHaven, & Mellor, 2018). Due to requirements from NIH and many peer-reviewed journals, it is increasingly common for scientist conducting clinical trials research to pre-register their studies, though such pre-registration often involves describing study conditions and intended outcome variables, but not the specifics of the planned analyses (Tan et al., 2019). Such trial pre-registration is associated with a substantial decrease in likelihood of statistically significant primary outcomes, reinforcing the value of pre-registration for avoiding publication bias (Kaplan & Irvin, 2015). Beyond clinical trials, pre-registration has been an increasing focus of attention in social science research and is becoming common in some disciplines, such as psychology (Nosek & Lindsay, 2018). Notably, in some cases a study is registered after the study is complete, which does not provide clarity on whether analyses presented were pre-planned or not. Herein, we use the term ‘registered’ when it is not known if a study was pre-registered or ‘post-registered,’ or if referring to multiple studies where some were pre-registered and some were post-registered. It is currently unclear how common pre-registration and post-registration is in behavioral medicine, particularly outside of clinical trials. In studies that are registered, it is also unknown how clearly authors specify which analyses were registered.

Systematically examining reporting practices can help characterize how consistently and clearly behavioral medicine articles are providing information that helps readers evaluate research claims. A study by Riehm, Azar, and Thombs (2015) audited randomized controlled trials from top behavioral medicine journals published in 2013 and 2014 to examine reporting practices of randomized clinical trials published in those journals. They found that only one-third of articles clearly declared primary or secondary outcomes, and only half had been registered prior to study enrollment.

In the current study, we examined reporting practices in hypothesis-testing manuscripts (both randomized trials and other designs) published in 2018 in the four behavioral medicine journals with the highest impact factors that year. We aimed to describe the proportions of articles published in these journals in 2018 that provide transparency on several aspects of the analyses presented. We first looked at how clearly the articles presented whether the hypotheses, outcomes, and/or analyses were described as primary or secondary in regards to the overall data collection effort. Second, we examined whether there were statements reporting that the study was registered in a publicly available repository. If a study was registered, we examined how clearly the authors specified which hypotheses or analyses were registered and whether registration occurred before or after the study started. Third, we examined whether ‘exploratory’ or related terms were used in relation to analyses or aims presented. Finally, we examined whether there was a statement describing power analyses, and clarity about whether power analyses were conducted before or after the study was conducted. A pre-registered secondary aim of the current study was to test the hypothesis that the portion of articles engaging in these practices was greater in 2018 than in 2008, given the increased attention to issues of transparency and replicability, particularly in behavioral science, over that 10 year period (Shrout & Rodgers, 2018).

## Materials and methods

### Journal and article selection

To select journals with the highest impact factor in behavioral medicine, we first created a comprehensive list of journals meeting the following criteria: (a) first published prior to 2007; (b) articles published address behavioral aspects of health and do not include unrelated topics; (c) articles published cover more than one sub-specialty of behavioral medicine (e.g. not all articles are related to only obesity, or only sleep). To develop the list of potential articles, we reviewed a list of all journals in the Web of Science journal categories of (a) public, environmental and occupational health, (b) psychology, general, (c) psychology, clinical; social sciences (interdisciplinary); (d) health care sciences and services; and (e) psychology applied. We also reviewed journals published by behavioral medicine societies (Freedland, 2019). After developing an initial list, we asked several colleagues to identify any missing journals. To determine whether journals met our criteria, we reviewed journal names and journal article titles, then, as needed, we examined journal article abstracts. The journals were then ranked according to 2018 impact factor published by Journal Citation Reports (Clarivate Analytics) and the journals with the top four impact factors were selected.

To achieve a random selection of articles within the four target journals, we used R programing language to randomly select among articles. During the coding process, we excluded articles that were systematic reviews/meta-analyses, presented only qualitative data, or presented only descriptive analyses.

### Data extraction and outcomes

For each article, we extracted the descriptive variables of journal title, journal year, and study design. Study designs were classified as experimental (which we defined as comparing outcomes between groups who were randomized to different conditions), observational (non-randomized), and measurement development/validation. If the study design was experimental, we documented whether the study was described as a randomized controlled trial.

A codebook for outcome variables was developed via an iterative process. We started with simple categories such as whether power analyses were present or not. We then had two members of the research team apply these codes to articles published in our target journals in 2017 (a year we did not plan to report on). At a series of meetings, we reviewed discrepant codes and determined we needed additional codes when the existing codes were inadequately or inaccurately capture the text. We developed a codebook that included specific instructions for applying codes. The final categories are as follows (also listed in Table 1, first column). First, we coded whether the article explicitly described the outcomes, analysis, or hypotheses presented in the manuscript as primary or secondary. The goal was to ascertain whether the hypotheses, reported outcomes, and analyses were the primary purpose of the reported data collection effort, or not. Specifically, articles were coded as (a) explicitly describing the hypothesis/outcome/analysis as ‘primary’ (e.g. ‘The primary outcome was whether patients agreed to be tested.’ Carey, Coury-Doniger, Senn, Vanable, & Urban, 2008); (b) explicitly describing results as ‘secondary’ (e.g. ‘This secondary analysis of de-identified data was exempt from IRB review.’ Sharapova, Singh, Agaku, Kennedy, & King, 2018); (c) presenting clear evidence that results were secondary but without explicitly describing it as ‘secondary’ (e.g. article stated that analyses used data from a larger study, but did not use term ‘secondary.’ Zhao, Okoro, Li, & Town, 2018); or (d) not having clear evidence of being primary or secondary (i.e. did not use either term, and there was no indication that data used had been previously analyzed or collected for a different primary purpose; e.g. Donenberg, Emerson, & Kendall, 2018).

**Table 1.** Transparency characteristics of behavioral medicine journal articles published in four journals in 2008 and 2018.

	2008 articles (*n*=120), *N*(%)	2018 articles (*n*=162) *N*(%)	Chi-squared^a^	*p*-value
**Study Design**				
Experimental	29 (24.2)	20 (12.4)	7.66	.02
Observational	89 (74.2)	141 (87.0)		
Measure development/ validation	2 (1.6)	1 (0.6)		
**Description of hypotheses/outcomes/analyses as primary or secondary**				
Explicitly described as primary	9 (7.5)	4 (2.5)	9.65	.008
Explicitly described as secondary	3 (2.5)	22 (13.6)		
Evidence of being secondary, but not explicit	47 (39.2)	83 (51.2)		
Unclear whether primary or secondary	61 (50.8)	53 (32.7)		
**Study Registration**				
Registered	2 (1.7)	22 (13.6)	11.08	<0.001
Not registered	118 (98.3)	140 (86.4)		
**Use of exploratory terminology to describe analysis or aim**			0.95	0.62
Exploratory term used	35 (29.2)	51 (31.5)		
Exploratory term not used	85 (70.8)	110 (67.9)		
Exploratory factor analysis used	0 (0)	1 (0.6)		
**Power analysis**			<0.001	1.0
Power analysis for sample size, clearly prior to study	1 (0.8)	5 (3.1)		
Power analysis for sample size, suggestive of prior to study	7 (5.9)	7 (4.3)		
Power analysis for sample size, clearly post-hoc analysis	1 (0.8)	0 (0)		
Power analysis for sample size, unclear whether a priori or post-hoc	0 (0)	0 (0)		
Power analysis for effect size able to detect, given sample size	1 (0.8)	1 (0.6)		
No power analysis presented	110 (91.7)	149 (92.0)		

^a^ Chi-squared analyses for the variables ‘description of analyses as primary or secondary’ and for ‘power analysis’ were conducted using the transformed variables.

Second, we coded whether the authors included a statement that the study was registered in a public repository. If a study was registered, we coded whether it indicated which specific hypotheses/analyses were registered and whether registration occurred before or after the study started. Third, we coded whether the term "exploratory" or “post-hoc” was used in relation to analyses or aims or not, with a separate category for exploratory factor analyses.

Finally, we coded the content related to power analyses. Specifically, we coded whether there was (a) a power analyses for sample size that had strong language indicating it was conducted a priori (e.g. ‘prior to the study, we conducted … ’); (b) a power analyses for sample size that had language suggestive of being conducted a priori; (c) a power analyses for sample size that was clearly described as post-hoc; (d) a power analyses for sample size that was unclear whether it was a priori or post-hoc; (e) power analysis to determine effect size study would be able to detect, given the sample size; and (f) no power analyses presented. The final codebook is available in supplemental materials.

### Data extraction

After the codebook was finalized, two PhD students and one masters-level research associate (MLD, KC, and MCS) conducted the initial coding of articles, with each article coded by two researchers independently. Prior to coding, all three coders were trained by the study first author (MAM). During coding, coders had the option to select ‘unsure’ if they could not determine the correct code. Discrepancies between coders (including if one or more coder selected ‘unsure’) were discussed as a team. The final decision was made by a third coder (MAM). If the third coder was unsure or if she decided on a code that was not selected by the first two reviewers, a consultation occurred with a fourth coder (LS). Cohen’s kappa coefficients were computed for each variable based on the initial two coders, with and without excluding those that either coder indicated being unsure about.

### Analyses

The approach for coding articles and all analyses reported in this manuscript were pre-registered at OSF: osf.io/s4y2q. This is the primary report of this data set and no other reports are planned. All reported results reflect pre-planned analyses and no post-hoc analyses are reported. We aimed to analyze 173 articles from 2018 based on a power analyses for our primary aim of describing the portion of articles with outcomes of interest in 2018. Specifically, we set a goal of 90% confidence that we are within 5% of the population value of the descriptive outcome (e.g. proportion registered) in 2018, assuming that 20% or fewer have the outcomes of interest (which was a number selected based on our practice coding of 2017 articles). We sought to analyze a similar portion of total articles published in 2008 as 2018.

For our primary aim of describing current reporting practices, we performed descriptive analyses for the 2018 articles by reporting frequency of each code. For the aim of comparing outcomes between 2008 and 2018, we conducted a series of chi-squared test with a *p*-value of 0.05. If differences were found between 2008 and 2018, Fisher’s Exact Test for count data was used to determine which outcomes differed. Prior to these analyses, some variables were transformed into meaningful categories that captured the clarity of reporting, consistent with our pre-registered plan. Specifically, for the primary/secondary analysis variable, we combined into one group those that were explicit in being either primary or secondary. For the power analysis variable, we categorized manuscripts as (a) having no power analyses, (b) presenting power analyses that were ambiguous on timing; and (c) all other power analyses categories. In our pre-registration plan we also proposed to compare clarity of registration timing and specificity of registration across 2008 and 2018, however we felt there were too few studies with registration to conduct these analyses.

## Results

### 2018 Manuscript characteristics

The four journals that were selected are *American Journal of Preventive Medicine*, *Annals of Behavioral Medicine*, *Health Psychology*, and *Psychosomatic Medicine*. There were 516 total articles published in these journals in 2018, and we randomly selected and coded 183 articles. Of these, we excluded 16 due to being review papers and 5 due to lacking hypothesis testing. Thus, we include in these analyses 162 manuscripts from 2018. Of these 162 articles, 87% (*n* = 141) were observational design, 12.4% (*n* = 20) were experimental, and 0.6% (*n* = 1) were measurement design/validation. Of those that were experimental, 60% (*n* = 12) were described as randomized trials. For initial coding, with and without including the ‘unsure’ response in calculations, kappa coefficients were 0.88 and 0.90 for if study was registered, 0.60 and 0.67 for if study was primary/secondary, 0.61 and 0.84 for power analyses, and 0.64 and 0.78 for question about use of ‘exploratory’ language, respectively.

In 2018, explicit description of hypotheses/outcomes/analyses as primary was observed in 2.5% (*n* = 4) of articles and explicit description as secondary was observed in 13.6% (*n* = 22). An additional 51.2% (*n* = 83) of articles presented clear evidence that results were secondary but without use of term ‘secondary.’ A lack of any indicators of being primary or secondary was found in 32.7% (*n* = 53) of articles.

Study registration was reported in 13.6% (*n* = 22) of the articles examined and was not reported in the remaining 86.4% (*n* = 140). Of the 22 that were registered, 18.2% (*n* = 4) were unambiguously described as registered a priori, one was unambiguously described as registered post-hoc, and 77.3% (*n* = 17) were lacking clear description of when registration occurred. For 20 of the 22 (90.9%) studies reporting registration, it was unclear which specific analyses were registered; 1 (4.5%) study specified that all analyses were registered; and 1 (4.5%) study specified at least one analysis that was registered.

The use of ‘exploratory’ or ‘post-hoc’ to describe study analyses or aims was present in 31.5% (*n* = 51) of the manuscripts. The remaining 68.5% (*n* = 111) did not use these terms.

In 2018 articles, 8% (*n* = 13) of articles presented power analyses. Five of these (3.1% of all 2018 articles) included strong language indicating that the power analysis was conducted prior to the study, seven (4.3%) had language suggestive that it was conducted prior to the study (but not considered strong language), and one (0.6%) was described as an analysis to determine the effect size that could be detected, given the available sample size.

### Comparison of 2008 and 2018 manuscripts

There were 439 total articles published in our four target journals in 2008, and we randomly selected and coded 143 articles. We excluded 2 due to being exclusively qualitative, 16 due to being review papers, and 5 due to lacking hypothesis testing. Thus, we include in this analysis 120 manuscripts from 2008. Study characteristics are provided in Table 1. In comparison, a higher portion of articles were observational in 2018 than 2008. Articles published in 2008 and 2018 differed in how clearly hypotheses/outcomes/analyses were described as primary or secondary, with articles more likely to be considered unclear with regard to being primary or secondary in 2008 than in 2018 (OR: 0.47; 95% CI: 0.28–0.79). Study registration was more common in 2018 than 2008 (see Table 1. See supplemental text for how transparency characteristics differed across experimental and observational studies in 2008 and 2018).

## Discussion

When assessing the evidentiary value of behavioral medicine research findings, readers benefit from having knowledge of the context of the analyses presented, including if analyses were pre-planned or post-hoc. In the current study, we evaluated the extent to which hypothesis-testing articles published in high impact factor behavioral medicine journals are engaging in reporting practices that increase transparency of analyses in this respect. We found that approximately one-third of the 2018 studies examined did not have sufficient information for readers to determine whether the hypotheses, outcomes, or analyses presented were the primary or secondary purpose of the data collection effort, whereas another half of articles appeared to report secondary hypotheses/outcomes/analyses, but did not use the term secondary. Additionally, we observed that about one-third of studies used the terms exploratory or post-hoc to describe any analyses or aims. We also found that few behavioral medicine publications included registration, though registration was more common in 2018 than 2008. Further, when registration did occur, key information for interpreting registration was usually missing, including if the study was pre-registered or registered after study completion. Finally, we observed that only a small portion of studies reported power analyses. These results suggest that additional efforts are warranted to increase transparent reporting.

Strengths of this study include a systematic and pre-specified approach to identifying the highest impact factor behavioral medicine journals; an analysis plan that was pre-registered; and use of a systematic approach to coding analyses. This study also had limitations. In coding some of the variables, we relied on the presence or absence of key words to classify the certain characteristics (e.g. ‘exploratory’), and it is possible that some authors used less common phrasing to convey similar meaning. However, if uncommon phrasing is used, it may be less likely to be interpreted as intended by readers. Another limitation of this study is that we did not have adequate power to provide precise estimates of transparent practices for each design type, or adequate power to compare 2008 and 2018 separately for each design. Additionally, while we aimed for a sample size of 173 articles in 2018, due to more exclusions than anticipated, we ended up with fewer than this number. Another limitation of this study is that the reliability was low for some of the codes. Nonetheless, given our procedures that involved adjudication of inconsistent codes by two experienced researchers, we expect that the final codes are an accurate representation of the literature. The inconsistent coding observed may reflect the difficulty of interpreting the language used to describe the practices of many of the articles, which suggests the need for shared language and attention to reporting these details. Our analyses were not focused on examining variation in reporting across journals, and although all the journals included have a behavioral medicine focus, they differ in the types of study designs most commonly published and in the extent of content published that overlaps with other disciplines (e.g. *American Journal of Preventive Medicine* includes many public health focused manuscripts).

A previous audit of behavioral medicine manuscript reporting by Riehm et al. focused only on clinical trials, and similarly found that the majority of studies did not clearly report their outcomes were primary or secondary (Riehm et al., 2015). Riehm et al. also found that nearly half of trials were not registered; our study found that 65% of experimental studies were registered, while 6% of observational studies reported a registration. Unlike Riehm et al., the current study focused on both experimental and observational studies, and found that the majority of manuscripts presented in top behavioral medicine journals are observational and secondary, which potentially present different challenges with regard to reporting on the context of analyses examined herein.

It is notable that several transparency practices improved between 2008 and 2018, including study registration and clarity regarding outcomes, analyses, or hypotheses presented being primary or secondary. These trends may be due in part to increased use of reporting guidelines as recommended or required upon submission to many behavioral medicine journals. Greater registration may also be attributable to increased NIH requirements to pre-register clinical trials and increased focus in behavioral science more generally to the concept of study pre-registration for hypothesis testing studies across study design types (Nosek & Lindsay, 2018).

To continue to improve manuscript reporting of the context of analyses presented, there are steps that can be taken by authors, reviewers, academic societies, and journal editors. Potentially, authors should be encouraged or required to specifically state in their manuscripts whether analyses were pre-planned or post-hoc. Wider use of pre-registration would further allow for verification of claims of pre-specified analyses and, in our experience, can help researchers to become more aware of when analyses become exploratory. Journals may consider encouraging pre-registration by requiring transparency statements as suggested by the Transparency and Openness Promotion Guidelines (Nosek et al., 2015) or by using ‘open science badges’ to highlight pre-registered studies (Center for Open Science, 2020). Importantly, efforts are warranted to encourage authors to not just state that a study is registered, but to also be clear if a study was registered prior to a study or not (e.g. by using term ‘pre-registration’), and to specify which analyses presented were pre-registered and how analyses may have strayed from the a priori plan and why. Despite all the potential promise of pre-registration, it should be noted that some scientist argue that that pre-registration does not address the most pressing problems in psychological or behavioral science, and that a greater focus should be on improving theories and statistical modeling of theories (Szollosi et al., 2019)

Journals could also consider instituting a two-staged peer review process (also called ‘Registered Report’), in which journals agree in principle to accept a manuscript based on its proposed hypothesizes and methods prior to conduct of the study; such an approach reduces bias against null results (Chambers, 2019). Journals could also require and enforce authors’ adherence to appropriate journal reporting guidelines, such as the Journal Article Reporting Standards by APA (Appelbaum et al., 2018). Further, clinical trials registration could be expanded to include pre-registering statistical analyses plan in more detail (in addition to pre-registering the study design and primary/secondary outcomes). Academic societies can also play a role by offering trainings for their members on how to increase transparency in their science. Individual authors can improve their reporting by including critical information about analyses presented; using the present article as an example, we stated in the Methods section:

The approach for coding articles and all analyses reported in this manuscript were pre-registered at osf.io/s4y2q. This is the primary report of this data set and no other reports are planned. All reported results reflect pre-planned analyses and no post-hoc analyses are reported.

Hence, a great deal of clarity can be provided using very few words. A final recommendation is that future efforts to audit behavioral medicine reporting practices build on existing reporting guidelines and existing coding approaches (e.g. Riehm et al., 2015, the current manuscript) in order to standardize efforts to characterize manuscript reporting practices, including changes over time.

Critically, reforms that seek to improve pre-registration and reporting transparency must also be paired with greater acceptance of exploratory research results. If exploratory results cannot be published in high quality journals, then researchers might find it too risky to pre-register their analyses or report exploratory analyses transparently. In our view, exploratory research results can be highly generative and valuable when they are clearly labeled as such. Moreover, pre-registration does not prevent researchers from exploring their data, and instead serves as a reminder for authors, and evidence for readers, of which analyses were planned at the outset and which were data driven.

The majority of the studies examined were observational and secondary analyses. Whereas pre-registration has clear benefits for providing a verifiable documentation of the timing of study decisions in relation to data collection, the benefits of pre-registration becomes more debated when conducting secondary analyses of existing data sets. In particular, because some portion of the data has already been presented and because it can not be confirmed that the study team has not already explored the data set, pre-registration no longer serves to verify to readers that certain analyses were pre-planned and not data-driven (Burlig, 2018). Nonetheless, even with secondary analyses of existing data, registering an analyses plan prior to conducting an analysis can help keep a researcher focused and honest with themselves, even if it can not offer the same level of confidence to others. An alternative approach to addressing risk of Type I error in analyses of existing data sets is multiverse analyses (Simonsohn, Simmons, & Nelson, 2015; Steegen, Tuerlinckx, Gelman, & Vanpaemel, 2016).

The low frequency of power analyses observed in this study should be considered in light of the fact that a large portion of studies examined appeared to be secondary analyses of existing data sets, where sample size is already determined. Yet power analyses may still serve a purpose in these cases. Specifically, authors can conduct sensitivity analyses to determine the power that they have to detect an effect size that would be clinically or practically meaningful, given their existing sample size. (Note that this is different from the practice of taking an obtained effect size and using it in a power analyses, which is widely recognized as flawed.(Zhang et al., 2019)) In many cases, such analyses could be conducted prior to running the secondary analyses and inform the decision to proceed. Journals and reporting guidelines can aim to increase the appropriate use of power analyses by requiring a statement about sample size determination (including statistical power, when appropriate), as is already required by some high impact behavioral science journals (Association for Psychological Science, 2020) and reporting guidelines (Schulz et al., 2010; von Elm et al., 2008).

Of these recommendations, pre-registration requires the greatest forethought on the part of authors and is likely to meet the most resistance. Indeed, even though we are proponents of pre-registration, it has taken time for us to learn to practice pre-registration consistently and well. Luckily, there are a now variety of resources available to help researchers pre-register their studies, such as AsPredicted.org and the Open Science Framework, which each provide pre-registration templates. In our experience, specifying analyses in advance of data collection and can streamline manuscript preparation, raise analytic complexities at a time when such insights can prevent critical problems, and can also be extremely helpful for training students. Hence, while there is a learning curve for pre-registration, we have found it well worth the effort.

## Conclusions

These results indicate that there are a variety of ways in which scientific reporting practices in behavioral medicine could improve. It is often unclear whether a given article is reporting primary versus secondary outcomes, or whether the reported analyses were planned in advance or were exploratory and data driven. Additionally, study registration is rare, and when used, reporting often lacks key details, and power analyses are also uncommon. Additional work may be warranted to develop and dissemination best practices for pre-registration and power analyses for secondary analyses in behavioral medicine, in particular. Support from journals for work that is explicitly exploratory may also improve transparency in reporting. Greater attention to reporting these details could improve interpretability and reliability of the health behavior literature.

## Supplementary Material

Supplemental Material

## Data statement

Dataset and statistical code for these analyses are available at the OSF page for this project: https://osf.io/s4y2q/
