# Supplementary material for: Transparent reporting of hypotheses and analyses in behavioral medicine research: an audit of publications in 2018 and 2008
Source: Health Psychol Behav Med. 2021 Apr 7;9(1):285–97. doi: 10.1080/21642850.2021.1907186 (PMC8145985; doi:10.1080/21642850.2021.1907186)
Supplement: Supplemental Material [file RHPB_A_1907186_SM0868.docx]

**Supplemental material.**

1. Coding instructions used

2. Supplemental table 2

**CODING for BMED Open Science review**

1. Name of coder______________
2. Date of coding_______________
3. Full reference (Vancouver or AMA style):
4. Select which year: 2008 2018
5. Select which journal: (list out final four; for now label as 1-4 and we’ll add titles later
6. Is the manuscript a Meta-analysis or review?
   1. Response options
      1. Yes (exclude study from remaining questions
      2. No
      3. Unsure. Explain ____________________________
   2. Instructions
      1. If it has term “review” or meta-analysis in title. Skim abstract to confirm.
7. Does the manuscript present qualitative data in absence of any quantitative data
   1. Response options
      1. Yes (only qual data) *(exclude from remaining questions)*
      2. No
      3. Unsure. Explain_______________________________
   2. Instructions
      1. Search for term “qualitative.” It should be in title or abstract. Skim abstract to determine if there might also be quantitative data (beyond reporting of participant characteristics).
8. Does article propose to test a hypothesis vs being purely descriptive?
   1. Response options
      1. Yes (hypothesis test is presented)
      2. No *(exclude from remaining questions)*
      3. Unsure. Explain_______________________________
   2. Instructions
      1. Read the final paragraph(s) of the introduction. This should include a description of the purpose of the study. Look to see if their purpose indicates that they are making a comparison or testing a hypothesis, vs “describing” or “characterizing”.
9. Which best describes the study design?
   1. Response options:
      1. Experimental/random assignment to a condition
      2. Observational
      3. Scale development and/or validation study
      4. Unsure. Explain: _______________________
   2. Instructions
      1. Search for “random”
      2. If participants were randomly assigned to different conditions and the aim of the study is on observing how the randomly assigned conditions differ code it as experimental. This could be between subjects (half randomized to one condition) or within subjects (complete two different tasks, with order randomized). Read aims and methods section, as needed. Note that some studies include randomization but they are not primarily looking at effects of randomization on outcomes.
      3. Scale development or validation study should describe in the title that it was focused on developing or testing a scale/measure.
      4. Everything else should be observational. (note, if the main focus of a study is mediation, it is observational, even if there is randomization within the study)
      5. If there is more than one type of study design included in one report, consider the one that is described as primary. If none described as primary, focus on first hypothesis/aim presented.
10. (*if experimental*): Did authors describe their study as an RCT?
    - 1. Yes
      2. No
    1. Instructions:
       1. Search for term “trial.” Code If the study is described anywhere as a “randomized controlled trial” or “randomized clinical trial” or a “randomized trial,” code it as an RCT. (code also for British spelling)
11. Was it reported that any aspect of the study was registered on public site?
    1. Response options:
       1. Yes (there was indication of registration of some part of study)
       2. No
       3. Unsure. Explain: ____________________________
    2. instructions
       1. To determine this, search for the term “regist” and “clinicaltrials.gov” Be sure that your search would pick up things that are not in main body of text but are in notes/acknowledgement. If there is any mention that study was registered or has a registration or is listed on clinicaltrials.gov, mark as yes
12. If registered, paste registration details here (registration location, reg #):
13. *If registered*: Was it clear which specific hypothesis/analyses were pre-registered?
    1. Response options:
       1. At least one hypothesis reported in present article was clear about being pre-registered, but not all were clear
       2. All were clear
       3. None were clear
       4. Unsure. Explain: ____________________________
    2. Instructions:
       1. Use search terms above. Where mention of registration was, is there any mention of which analyses were registered?
       2. Skim the methods and results to see if there is any mention of a specific analyses being registered.
14. If registered, was the article explicit about if registration was before or after study started/participant enrollment?
    1. Response options

i. yes: explicit that it was registered before study started

ii. yes: explicit that it was not registered before study started

iii. no: it was not explicitly stated if it was registered before or after

iv. unsure. Explain:___________________________________________________

- 1. Instructions
     1. Code #1 if they call it "pre-registration" or otherwise state that registration occurred prior to study start or participant enrollment.
     2. Code #2 if they indicate that it was registered, but explicitly state that it was done after data collection started. (e.g., describe registration as retrospective)

1. Is it clear if the analyses being presented were the primary or secondary analyses of the data collection effort?
   1. Response options:
      1. Uses the term “primary” to describe purpose/outcomes of data collection effort.
      2. Uses the term “secondary” to describe the entirety of the data presented.
      3. Doesn’t use term secondary, but clearly is NOT presenting the primary purpose/outcomes
      4. It is unclear if it is primary or secondary
      5. I am not sure. Explain: ______________
   2. Instructions:
      1. Search for term “primary,” “second*”, “Published”, “previously”, “original” and “parent” and read the context of any thing you find. Additionally, read following section to see if there is any mention of other publications using same data set: last paragraph of intro, beginning of the methods (often the “design” section), participants section of methods.
      2. For “clearly primary” they should use the term “primary” in regards to the purpose/aim/hypothesis of the trial/project/study. It is not enough to just say “primary aims” “primary hypothesis” or “primary purpose” because that could just be the aims of the current manuscript—we need a statement that indicates that it was the primary results from the data collection effort. To code as “clearly primary” there should also be an absence of any of the above indicators that this was secondary (as described below).
      3. Search for “second*”. To code as secondary, needs to use the term secondary to describe the entirety of the project. For example, they might say that it was a secondary analysis of a RCT.
      4. To code as “Doesn’t use term secondary, but clearly is NOT presenting the primary purpose/outcomes”: there needs to be a clear indication that there are outcomes/results from same data set published elsewhere (vs it being unclear if what they reference is just a protocol or not). This would also be coded if they indicate that it is a large, publically available data set (such as NHANES, HINTS).
      5. If none of above criteria are met code as “unclear.”
2. How was sample size/power analysis addressed with regard to the specific study sample analyzed in this manuscript?
   1. Response options:
      1. Power analysis presented using terms that inarguably indicate it was done prior to study (“a priori”; “prior to study”; “prior to study”)
      2. Power analysis presented using terms that are strongly suggestive it was done prior to study (e.g., via sentence structure, use of past tense).
      3. Power analysis presented, but it was ambiguous if it was done before or after the study started.
      4. Power analysis presented, but authors’ indicate that it was conducted post-hoc.
      5. Analysis conducted to determine what size of an effect could be detected, given a certain sample size (i.e., the sample they have access to; not used to plan sample size)
      6. No power analyses or effect size analyses for this study sample presented
      7. Unsure. Explain: _______________________________
   2. Instructions
      1. Search for “sample” “power” and “sensitivity analysis” Read participant and analysis sections.
      2. It is not enough if they said only that they did a power analyses. They need to have presented the details of the analyses to code any of above except vi.
      3. If they present power analyses result for a parent study but do not present power analyses specifically for this study, this would be coded as “no power analyses.”
3. Is there any reference to an analyses being conducted that was not planned in advance using terms “explor*” or “post hoc.”
   1. Response options:
      1. Yes, term used to describe a study analysis or aim; (if only used in “exploratory factor analysis” do not endorse)
      2. “Exploratory factor analysis” included but no other use of these terms.
      3. No use of these terms
      4. unsure. Explain: ______________________
   2. Instructions
      1. Search for each term. If it is used, see if it is being used to describe an analysis or a research aim. Verify that it is being used in a way that clearly indicates to readers that this was an exploratory or unplanned analysis.
      2. Do not count if the reference is only to them doing a post-hoc analysis on an anova (such as Tukey HSD), as this is not really relating to the entirety of the analysis.

14. Provide additional notes here or if there are interesting examples that we may want to cite in the paper, provide them here (mention why they are interesting)

**SUPPLEMENTAL Table 2**. Transparency characteristics in 2008 and 2018 behavioral medicine peer-reviewed journal articles, by study design.

|  | 2008 articles | | 2018 articles | |
| --- | --- | --- | --- | --- |
| Outcome | Experimental (n=29)  N(%) | Observational (n=89)  N(%) | Experimental (n=20)  N(%) | Observational (n=141)  N(%) |
| **Description of analyses presented as primary or secondary** |  |  |  |  |
| Explicitly described as primary | 7 (24.1%) | 1(1.1%) | 2 (10.0) | 2 (1.4) |
| Explicitly described as secondary | 0 (0%) | 3 (3.3%) | 2 (10.0) | 19 (13.5) |
| Evidence of being secondary, but not explicit | 20 (69.0%) | 40 (44.9%) | 11 (55.0) | 42 (29.8) |
| Unclear whether primary or secondary | 2 (6.9%) | 45 (50.6%) | 5 (25.0) | 78 (55.3) |
| **Registration** |  |  |  |  |
| Registered | 2 (6.9%) | 0 (0%) | 13 (65.0) | 9 (6.4) |
| Not registered | 27 (93.1%) | 89 (100%) | 7 (35.0) | 132 (93.6) |
| **Use of exploratory terminology to describe analysis or aim** |  |  |  |  |
| Exploratory term used | 6 (20.7%) | 28 (31.5%) | 6 (30.0) | 45 (31.9) |
| Exploratory term not used | 23 (79.3%) | 61 (68.5%) | 14 (70.0) | 95 (67.4) |
| Exploratory factor analysis used | 0 (0%) | 0 (0%) | 0 (0) | 1 (0.7) |
| **Power analysis** |  |  |  |  |
| Power analysis for sample size, clearly prior to study | 1 (3.4%) | 0 (1.1%) | 3 (15.0) | 2 (1.4) |
| Power analysis for sample size, suggestive of prior to study | 6 (20.7%) | 1 (1.1%) | 4 (20.0) | 3 (2.1) |
| Power analysis for sample size, clearly post-hoc analysis | 0 (0%) | 1 (1.1%) | 0 (0%) | 0 (0%) |
| Power analysis for sample size, unclear whether a priori or post-hoc | 0 (0%) | 0 (0%) | 0 (0%) | 0 (0%) |
| Power analysis for effect size able to detect, given sample size | 1 (3.4%) | 0 (0%) | 0 (0) | 1 (0.7) |
| No power analysis presented | 21 (72.4%) | 87 (97.8%) | 13 (65.0) | 135 (95.7) |
